# Characteristics and critical care interventions in drowning patients treated by the Danish Air Ambulance from 2016 to 2021: a nationwide registry-based study with 30-day follow-up

**DOI:** 10.1186/s13049-024-01189-y

**Published:** 2024-03-06

**Authors:** Niklas Breindahl, Signe A. Wolthers, Thea P. Møller, Stig N. F. Blomberg, Jacob Steinmetz, Helle C. Christensen, the Danish Drowning Validation Group

**Affiliations:** 1 Prehospital Center Region Zealand, Ringstedgade 61, 13, 4700 Næstved, Denmark; 2 Department of Clinical Medicine, University of Copenhagen, Copenhagen, Denmark; 3 Department of Neonatal and Paediatric Intensive Care, Copenhagen University Hospital, Rigshospitalet, Blegdamsvej 9, 2100 Copenhagen, Denmark; 4 Department of Anesthesiology and Intensive Care Medicine, Holbæk Hospital, Holbæk, Region Zealand Denmark; 5 Danish Air Ambulance, Brendstrupgårdsvej 7, 8200 Aarhus, Denmark; 6 Department of Anaesthesia, Centre of Head and Orthopaedics, Rigshospitalet, Blegdamsvej 9, 2100 Copenhagen, Denmark; 7 Faculty of Health, Aarhus University, Aarhus, Denmark

**Keywords:** Drowning, Danish drowning formula, Danish Air Ambulance, Helicopter emergency medical service (HEMS), Emergency medical service (EMS), Registry-based, National Danish Drowning Registry

## Abstract

**Background:**

Improving oxygenation and ventilation in drowning patients early in the field is critical and may be lifesaving. The critical care interventions performed by physicians in drowning management are poorly described. The aim was to describe patient characteristics and critical care interventions with 30-day mortality as the primary outcome in drowning patients treated by the Danish Air Ambulance.

**Methods:**

This retrospective cohort study with 30-day follow-up identified drowning patients treated by the Danish Air Ambulance from January 1, 2016, through December 31, 2021. Drowning patients were identified using a text-search algorithm (Danish Drowning Formula) followed by manual review and validation. Operational and medical data were extracted from the Danish Air Ambulance database. Descriptive analyses were performed comparing non-fatal and fatal drowning incidents with 30-day mortality as the primary outcome.

**Results:**

Of 16,841 dispatches resulting in a patient encounter in the six years, the Danish Drowning Formula identified 138 potential drowning patients. After manual validation, 98 drowning patients were included in the analyses, and 82 completed 30-day follow-up. The prehospital and 30-day mortality rates were 33% and 67%, respectively. The National Advisory Committee for Aeronautics severity scores from 4 to 7, indicating a critical emergency, were observed in 90% of the total population. They were significantly higher in the fatal versus non-fatal group (p < 0.01). At least one critical care intervention was performed in 68% of all drowning patients, with endotracheal intubation (60%), use of an automated chest compression device (39%), and intraosseous cannulation (38%) as the most frequently performed interventions. More interventions were generally performed in the fatal group (p = 0.01), including intraosseous cannulation and automated chest compressions.

**Conclusions:**

The Danish Air Ambulance rarely treated drowning patients, but those treated were severely ill, with a 30-day mortality rate of 67% and frequently required critical care interventions. The most frequent interventions were endotracheal intubation, automated chest compressions, and intraosseous cannulation.

## Background

Drowning is defined by the World Health Organisation (WHO) as "the process of experiencing respiratory impairment from submersion or immersion in liquid” [1]. Drowning is a time-critical event, and hypoxia is the cornerstone of drowning pathophysiology [2–4]. Early optimisation of oxygenation and ventilation is paramount in managing drowning [5]. Drowning often occurs in remote locations with prolonged transport times to the hospital, emphasising the need for early prehospital treatment. Therefore, Emergency Medical Service (EMS) personnel should master drowning management and apply appropriate critical care interventions early in the prehospital setting.

In 2014, the Danish EMS introduced a national physician-staffed helicopter emergency medical service (HEMS, the Danish Air Ambulance) to ensure advanced critical care, including advanced airway management, early in the prehospital setting for all patients regardless of geographical location on the ground [6]. Prehospital interventions and outcomes have previously been evaluated in paediatric drowning managed by physician-staffed HEMS, showing a high requirement for prehospital critical care interventions in decentralised areas and transfer to paediatric specialist centres [7]. However, there is little information on the interventions and outcomes for the nationwide population of fatal and non-fatal drowning across all age groups treated by a physician-staffed HEMS. The population of drowning patients treated by the Danish Air Ambulance has never been described, thus contributing to the potential underreporting of the most critically ill drowning patients in Denmark.

Given the rarity of drowning, registry-based studies are needed to provide patient characteristics and evaluate critical care interventions performed on this group of patients.

## Methods

### Aim

This study aimed to describe patient characteristics, 30-day mortality, and critical care interventions in drowning patients treated by the Danish Air Ambulance.

### Study design

We conducted a nationwide registry-based study with a 30-day follow-up. Data on fatal and non-fatal drowning missions between 2016 and 2021 were derived from the Danish Air Ambulance database.

### Setting

The Danish National Health Services provides all citizens with free and universal tax-supported health care [6]. Prehospital healthcare is divided into five regions, each with a regional Emergency Medical Dispatch Centre [8]. The Danish Air Ambulance is a Helicopter Emergency Medical Service (HEMS), which is part of the governmentally funded EMS and operates round-the-clock. It is staffed by a consultant-level anaesthesiologist with extensive prehospital experience, a HEMS crew member being an experienced paramedic with supplemental training, and a pilot. The Danish Air Ambulance may be dispatched for time-critical events at locations on the ground with prolonged transport time by the medical dispatchers (specifically trained healthcare professionals) to provide advanced patient care on-scene and during transport to specialised care [6]. The Royal Danish Air Force’s Search And Rescue helicopters are used for missions at sea. These missions are described elsewhere and were not included in this study [9]. The Danish Air Ambulance is dispatched following a criteria-based protocol based on (1) the 1–1–2 call, (2) on-scene crew request, (3) inter-hospital transfers, and (4) non-critical missions to smaller islands not connected by road to the mainland [8, 10]. The dispatch criteria include major trauma and mass casualty, drowning, diving incidents, and time-critical conditions such as acute myocardial infarction, out-of-hospital cardiac arrest with return of spontaneous circulation (OHCA with ROSC), and stroke. On October 1, 2014, the Danish Air Ambulance became national, with three helicopter bases to ensure geographical coverage within approximately 30 min response time. On January 2, 2019, a fourth helicopter base was placed in Northern Denmark [11].

### The Danish Air Ambulance database

The Danish Air Ambulance database was established in 2014. It contains aggregated, high-quality data for all missions on a national level and provides a solid basis for research and quality improvement [6].

Within hours after a patient encounter, the physician registers mission data and selects a primary and a secondary prehospital diagnosis code based on the International Classification of Diseases, 10th Edition, in the database. In addition to the predefined variables in the Danish Air Ambulance database, a note field is provided for a short, free-text medical report. Missing data accounts for less than 6.5% of most variables and misclassification is observed in less than 4% of missions [6].

### Participants

Breindahl et al. (2023) recently demonstrated that a text-search algorithm (Danish Drowning Formula) could search unstructured text fields of the electronic medical records in the Danish Cardiac Arrest Registry, which contains all recorded OHCA in Denmark, and identify drowning-related OHCA [12]. The Danish Air Ambulance also contains unstructured text fields and records all air ambulance missions in Denmark. Therefore, we used the Danish Drowning Formula to search for drowning incidents within the relevant primary and secondary diagnosis codes according to the WHO International Classification of Diseases (ICD) 10th Revision (DT751/drowning, DI460/cardiac arrest with ROSC, DI461/sudden cardiac death, and DT689/accidental hypothermia) in the Danish Air Ambulance database from January 1, 2016, through December 31, 2021. Two independent physicians manually reviewed all potential drowning patients identified by the Danish Drowning Formula for validation using the internationally accepted definition of drowning as “the process of experiencing respiratory impairment from submersion or immersion in liquid” supplemented with the non-fatal drowning categorization framework and clarification statement [1, 13]. This study used follow up at 30 days after the drowning incident to distinguish between fatal and non-fatal drowning. A senior consultant solved any discrepancies. We excluded duplets and interhospital transfers. All patients with a valid civil registration number completed the 30-day follow-up [14].

### Outcomes

The primary outcome was 30-day mortality in drowning patients treated by the Danish Air Ambulance. The secondary outcomes were prehospital mortality and the number and type of critical care interventions.

### Variables

The Danish Air Ambulance database variables include dispatching region, mission date and time, response time, on-scene time, total mission time, and prehospital diagnosis codes assigned by the physician [6]. Transport type was determined as airlifted by the Danish Air Ambulance, transported by ambulance with or without physician escort, non-conveyance, and patient declared dead on-scene.

Critical care interventions performed by the Danish Air Ambulance or ground-based EMS physician were endotracheal intubation, administration of blood products, intraosseous cannulation, use of an automated chest compression device, ultrasound examination, chest tube placement, and thoracostomy. Ultrasound examination was included as a critical care intervention in this study as it may distinguish between, or rule out, specific emergency conditions such as ruptured aortic aneurysm, acute cardiac failure, and pulmonary diseases. This may impact both prehospital treatment and decision-making in triage and transport [15, 16].

The National Advisory Committee for Aeronautics (NACA) severity score (range 0–7) correlates well with morbidity and mortality and was reported for all patients [17, 18]. NACA scores of four or above are considered to represent a patient with severe or critical illness or injury [10, 19].

Data from the Danish Patient Registry on 30-day mortality were linked to all included patients with a valid civil registry number (a unique identification number assigned to each Danish citizen).

Two independent physicians manually extracted data on the type of location and liquid from the reports based on the geographical coordinates and the note fields according to previously used definitions. The location type was categorised as (1) Bathtub, (2) Swimming pool (including all in- or outdoor structures designed to hold a body of standing water), (3) Harbour, (4) Coastline, and (5) Lake. The type of activity at the time of the incident was categorised as (1) Swimming/bathing (including all forms of playing, bathing, and swimming), (2) Boat activity (including activities on a boat in open water, such as oceans and lakes, including fishing from a boat), (3) Diving (including freediving and scuba diving), (4) Recreational fishing, (5) Traffic (including incidents involving driving into the water using a bike or motorised vehicle), (6) Harbour activity (including falling overboard while the boat was in port), (7) Water sports (including incidents involving swim practice, kayaking, water polo, and surfing).

Eurostat's Degree of Urbanization from 2012 was used to classify the area of drowning incidents by population density [20]. The classification was divided into three categories: densely populated areas (cities), intermediate-density areas (towns and suburbs), and thinly populated areas (rural areas).

### Statistical methods

Baseline characteristics were presented separately for fatal and non-fatal drowning according to 30-day mortality. Categorical variables were presented as counts and percentages and were compared using the chi-squared test. Non-parametric variables were presented as medians with interquartile ranges [IQR] and compared using the Mann–Whitney U test. P-values < 0.05 were considered statistically significant. Significant p-values for variables with more than two levels were followed by pairwise comparisons using Fisher’s exact test adjusted for multiple testing with the Holm method. There was no imputation of missing data. All analyses were performed using R statistical software (version 4.2.2) [21].

## Results

### Study population

**Fig. 1:**
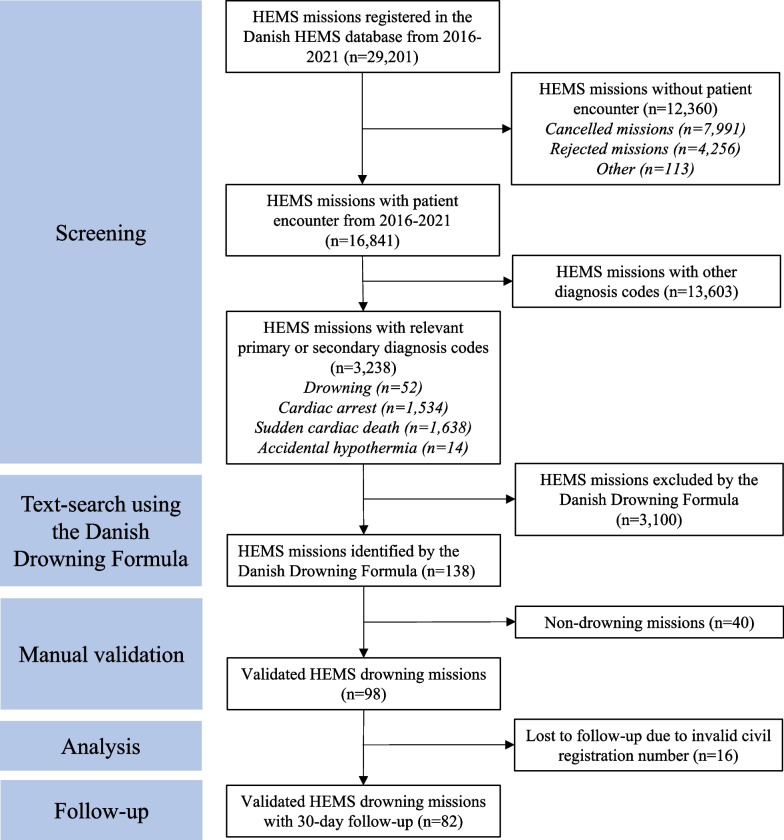
STROBE inclusion flow chart. Flow chart of screening, inclusion, exclusion and 30-day follow-up

**Fig. 2:**
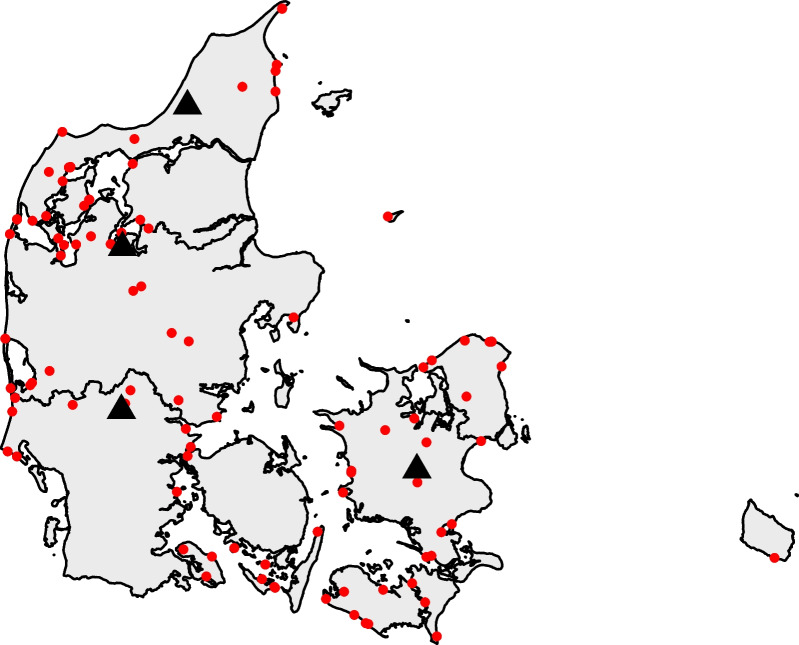
Map of drowning patients treated by the Danish Air Ambulance from 2016 to 2021. Red dots = drowning missions treated by the Danish Air Ambulance with a patient encounter. Black triangles = Danish Air Ambulance bases

From 2016 to 2021, the Danish Air Ambulance was dispatched 29,201 times (Fig. 1). We excluded 12,360 missions without patient encounters (7991 missions were cancelled, 4256 missions were rejected, and 113 missions did not result in a patient encounter for various other reasons). Within the remaining 16,841 missions with patient encounters, we identified 3238 missions with relevant diagnosis codes (1.6% DT751/drowning, 47% DI460/cardiac arrest with ROSC, 51% DI461/sudden cardiac death, and 0.4% DT689/accidental hypothermia) and excluded 13,603 HEMS missions with other diagnosis codes. The Danish Drowning Formula was applied on the 3238 missions and identified 138 missions, where the electronic medical records contained at least one trigger word from the text-search algorithm [12]. The remaining 3100 HEMS missions with relevant diagnosis codes did not contain any trigger words from the Danish Drowning Formula and were excluded. There were no duplets or interhospital transfers among the identified 138 patients. After manual validation, 40 HEMS missions were categorised as “non-drowning” and excluded. A total of 98 drowning patients were included in this study (Fig. 1), resulting in a positive predictive value of the Danish Drowning Formula = [98/(98 + 40)] × 100 = 71%. The location of all drowning missions treated by the Danish Air Ambulance are presented on a map of Denmark (Fig. 2).

### Operational data from the Danish Air Ambulance

The operational data from the Danish Air Ambulance are summarised in Table 1. There was no significant difference in total mission or response time between fatal and non-fatal drowning missions. The Danish Air Ambulance spent significantly more time providing medical care on-scene in the group with a fatal outcome (median [IQR] 28 [21–32] minutes vs. 19 [16–29] minutes, p = 0.01).

**Table 1:** Operational data of drowning patients treated by the Danish Air Ambulance from 2016–2021

	Non-fatal (n = 27)	Fatal (n = 55)	Total (n = 82)	p-value
Total mission time (min), median [IQR]	83 [54–101]	78 [55–112]	82 [55–107]	0.64
Response time (min), median [IQR]	20 [12–26]	21 [15–27]	21 [15–27]	0.42
On-scene time (min), median [IQR]	19 [16–29]	28 [21–32]	26 [19–30]	0.01*
*Danish Air Ambulance base, n (%)*				0.26
Saltum	3 (11)	6 (11)	9 (11)	
Billund	9 (33)	8 (15)	17 (21)	
Skive	8 (30)	21 (38)	29 (35)	
Ringsted	7 (26)	20 (36)	27 (33)	
*Population density at site, n (%)*				0.77
Thinly populated area	18 (67)	36 (65)	54 (66)	
Intermediate density area	9 (33)	17 (31)	26 (32)	
Densely populated area	0 (0)	1 (2)	1 (1)	
Missing	0 (0)	1 (2)	1 (1)	
*Season, n (%)*				0.16
Winter (Dec.–Feb)	2 (7)	7 (13)	9 (11)	
Spring (Mar.–May)	3 (11)	12 (22)	15 (18)	
Summer (Jun.–Aug.)	19 (70)	24 (44)	43 (52)	
Autumn (Sep.–Nov.)	3 (11)	12 (22)	15 (18)	
*Weekday, n (%)*				0.45
Monday–Friday	22 (81)	39 (71)	61 (74)	
Saturday–Sunday	5 (19)	16 (29)	21 (26)	
*Location, n (%)*				0.05
Bathtub	3 (11)	1 (2)	4 (5)	
Swimming pool	5 (19)	4 (7)	9 (11)	
Harbour	5 (19)	15 (27)	20 (24)	
Coastline	6 (22)	26 (47)	32 (39)	
Lake	3 (11)	4 (7)	7 (9)	
Missing	5 (19)	5 (9)	10 (12)	
*Liquid, n (%)*				< 0.01*^,a^
Freshwater	6 (22)	8 (15)	14 (17)	
Saltwater	10 (37)	41 (75)	51 (62)	
Chlorinated water	5 (19)	2 (4)	7 (9)	
Other	1 (4)	0 (0)	1 (1)	
Missing	5 (19)	4 (7)	9 (11)	
*Activity, n (%)*				0.84
Swimming/bathing	13 (48)	20 (36)	33 (40)	
Boat activity	1 (4)	1 (2)	2 (2)	
Recreational fishing	0 (0)	1 (2)	1 (1)	
Traffic	0 (0)	2 (4)	2 (2)	
Harbour activity	3 (11)	6 (11)	9 (11)	
Water sports	1 (4)	2 (4)	3 (4)	
Missing	9 (33)	23 (42)	32 (39)	

Information on 30-day follow-up was missing for 16 patients who survived prehospital due to missing CPR number. All analyses are unadjusted.

*IQR* Interquartile range, *min* minutes. Missing data are excluded from the denominator

*Statistically significant

^a^Significant p-value followed by pairwise comparisons using Fisher's exact test adjusted for multiple testing with the Holm method, resulting in no significant differences.

Most drowning missions treated by the Danish Air Ambulance occurred during the Danish summer months and public holidays of June–August (52%). However, no differences in mortality were observed for seasonal or weekday variation. The most frequent drowning locations for patients treated by the Danish Air Ambulance were coastlines (39%), harbours (24%), and swimming pools (11%) in decentralised geographical areas of Denmark with low population density (Fig. 2). Geographical location and population density were not significantly associated with mortality. The analysis showed an overall significant difference in mortality according to the type of liquid in which the drowning occurred, but pairwise comparisons adjusted for multiple testing showed no significant differences. The main activity prior to drowning was swimming/bathing (40%), followed by harbour activity (11%), where the patient most likely entered the water by accident. However, the type of activity was missing in the medical records in 39%.

### Medical data from the Danish Air Ambulance

The medical data from the Danish Air Ambulance are summarised in Table 2. Drowning was registered as the primary diagnosis in 45% and as the secondary diagnosis in 23%. A total of 26 patients (32%) did not have a primary or secondary diagnosis code of drowning. Other ICD-10 diagnosis codes represented in this population were DG40 (epilepsy), DT599 (intoxication), DJ939 (pneumothorax), and DT093 (traumatic spinal cord injury). Most drowning patients were male (62%). The median [IQR] age was 50 [18–69] years, and patients were significantly older in the fatal group compared to the non-fatal group (median [IQR] 58 [37–71] vs. 23 [6–53], p < 0.01). However, the age distribution showed a bimodal pattern, concentrating on ages one to six and beyond 60 years. For the paediatric subpopulation under six years, 75% were alive 30 days after the incident, compared to only 22.7% of individuals beyond 60 years.

**Table 2:** Medical data of drowning patients treated by the Danish Air Ambulance from 2016 to 2021

	Non-fatal (n = 27)	Fatal (n = 55)	Total (n = 82)	p-value
Sex, male, n (%)	19 (70)	32 (58)	51 (62)	0.42
Missing	0 (0)	16 (29)	16 (20)	
Age, years, median [IQR]	23 [6–53]	58 [37–71]	50 [18–69]	< 0.01*
*Age groups, n (%)*				0.06
< 1 years	1 (4)	0 (0)	1 (1)	
1 to < 6 years	5 (19)	2 (4)	7 (9)	
6 to < 14 years	5 (19)	1 (2)	6 (7)	
14 to < 19 years	1 (4)	3 (5)	4 (5)	
19 to < 26 years	2 (7)	1 (2)	3 (4)	
26 to < 46 years	4 (15)	5 (9)	9 (11)	
46 to < 60 years	4 (15)	9 (16)	13 (16)	
> 60 years	5 (19)	17 (31)	22 (27)	
Missing	0 (0)	17 (31)	17 (21)	
*Primary diagnoses, n (%)*				< 0.01*
DG40 Epilepsy	1 (4)	0 (0)	1 (1)	
DI460 Cardiac arrest with ROSC	6 (22)^b1^	10 (18)	16 (20)	
DI461 Sudden cardiac death	0 (0)^b2^	25 (45)^b1^	25 (30)	
DT751 Drowning	18 (67)	19 (35)^b2^	37 (45)	
DT599 Intoxication	1 (4)	0 (0)	1 (1)	
DT689 Accidental hypothermia	1 (4)	1 (2)	2 (2)	
*Secondary diagnoses, n (%)*				0.12
DI460 Cardiac arrest with ROSC	2 (7)	1 (2)	3 (4)	
DI461 Sudden cardiac death	0 (0)	1 (2)	1 (1)	
DJ939 Pneumothorax	0 (0)	1 (2)	1 (1)	
DT093 Traumatic spinal cord injury	1 (4)	0 (0)	1 (1)	
DT751 Drowning	2 (7)	17 (31)	19 (23)	
DT689 Accidental hypothermia	0 (0)	3 (5)	3 (4)	
None	22 (81)	32 (58)	54 (66)	
NACA score, median [IQR]	4 [4–5]	6 [6–7]	6 [5–7]	< 0.01*
NACA score > 3, n (%)	20 (74)	54 (98)	74 (90)	< 0.01*
*Critical care interventions, n (%), missing*				
At least one critical care intervention	13 (48), 0	43 (78), 0	56 (68)	0.01*
Endotracheal intubation	13 (48), 0	36 (65), 3	49 (60), 3	0.11
Intraosseous cannulation	4 (15), 2	27 (49), 0	31 (38), 2	0.01*
Automated chest compression device	2 (7), 2	30 (55), 2	32 (39), 4	< 0.01*
Ultrasound examination	6 (22), 2	22 (40), 2	28 (34), 4	0.21
Chest tube placement/thoracostomy	0 (0), 2	2 (4), 2	2 (2), 4	0.83
*Transport type, n (%)*				< 0.01*^,c^
Airlifted by the Danish Air Ambulance	15 (56)	24 (44)	39 (48)	
Transported by ambulance with physician escort	5 (19)	1 (2)	6 (7)	
Transported by ambulance without physician escort	5 (19)	2 (4)	7 (9)	
Non-conveyance	1 (4)	0 (0)	1 (1)	
Patient declared dead prehospital	0 (0)	27 (49)	27 (33)	
Missing	1 (4)	1 (2)	2 (2)	

Information on 30-day follow-up was missing for 16 patients who survived prehospital due to missing CPR number. All analyses are unadjusted.

*IQR* Interquartile range, *NACA* The National Advisory Committee for Aeronautics (NACA) severity score, *OHCA* Out of Hospital Cardiac Arrest, *ROSC* Return Of Spontaneous Circulation, *min* minutes. Missing data are excluded from the denominator

*Statistically significant

^b1−b1^ and ^b2−b2^ = significant p-value followed by pairwise comparisons using Fisher's exact test adjusted for multiple testing with the Holm method, resulting in significant differences (p < 0.05)

^c^ = No significant pairwise associations were observed after removing the "Declared dead on-scene" category

Most drowning patients treated by the Danish Air Ambulance (90%) were critically ill/injured, with NACA scores > 3 (98% vs. 74% in the fatal vs non-fatal group, p < 0.01).

A total of 39 patients (48%) were airlifted, but no significant associations between transport type and 30-day mortality were observed after removing the category "Patient declared dead prehospital".

### Outcome data

A total of 27 patients (33%) were declared dead prehospital, and 55 patients (67%) were dead at 30-day follow-up. At least one critical care intervention was performed in 68% of the drowning missions by a physician from either the Danish Air Ambulance or a physician-staffed ground EMS unit. Significantly more interventions were performed in the fatal group compared to the non-fatal group (78% vs. 48%, p = 0.01). There was no significant difference in the incidence of endotracheal intubation between the fatal and the non-fatal group (65% vs. 48%, p = 0.11). Intraosseous cannulation (49% vs. 15%, p = 0.01) and automated chest compressions (55% vs. 7%, p < 0.01) were performed significantly more in the fatal group.

## Discussion

### Summary of findings

This study is the first to describe a national cohort of fatal and non-fatal drowning patients treated by the Danish Air Ambulance. The population had high prehospital and 30-day mortality rates of 33% and 67%, respectively, and a high incidence of prehospital critical care interventions, particularly endotracheal intubation, use of an automated chest compression device, intraosseous cannulation, and ultrasound examination.

### Results compared to the existing literature

The available literature on a nationwide population of drowning patients treated by a physician-staffed HEMS is scarce. The available body of evidence focusing on physician-staffed HEMS mainly constitutes data on traumatic injuries, paediatric emergencies, cardiac arrests, or winch operations [7, 22–26].

The drowning population treated by the Danish Air Ambulance had a median age of 50 years with a bimodal pattern concentrating on ages one to six and beyond 60 years. For the paediatric subpopulation below 6 years, the 30-day survival was 75% compared to only 22.7% in the elderly individuals above 65 years. The improved survival rates in children are consistent with other studies of paediatric emergencies [7, 27, 28], and drowning-related OHCA in children may be correlated with a higher rate of bystander first aid and CPR [29, 30]. Also, one study indicated that paediatric drowning events were more likely to occur in swimming pools [7], which is associated with improved survival [12]. Nevertheless, drowning remains a frequent cause of HEMS dispatch in children and a leading cause of death in children [22, 31].

Comparisons between patient populations assessed by a physician-staffed HEMS and ground-based EMS may be inappropriate due to case-mix differences in illness severity and diagnoses, dispatch criteria, and skills of the medical staff [22].

Proper dispatch of HEMS is essential to reduce over-triage and costs. Patients transported by ambulance without HEMS physician escort with NACA scores less than three have previously been considered to indicate HEMS over-triage [10]. On the contrary, a high NACA score and the need for critical care interventions may indicate the relevance of HEMS dispatch [32]. Drowning patients treated by the Danish Air Ambulance were critically ill with high NACA scores more than three and a high incidence of endotracheal intubation and other critical care interventions. As previously demonstrated, patients were predominantly assessed in decentralised areas where helicopter dispatch most likely decreased the transport time compared to ground-based EMS [33, 34]. Yet, the median response time in this study was 21 min, emphasising the critical role of bystanders and trained lifeguards to assist with early rescue and lifesaving treatment [35, 36]. The results from this study indicate proper dispatch of the Danish Air Ambulance for the subpopulation of drowning patients with overall high NACA scores, decentralised geographical locations with prolonged transport times, and a high incidence of prehospital interventions.

As a surrogate measure of severity, we used the number of critical care interventions performed by the physician-staffed Danish Air Ambulance. More than two-thirds of the missions conducted by the Danish Air Ambulance resulted in at least one critical care intervention, particularly endotracheal intubation, use of an automated chest compression device, intraosseous cannulation, and ultrasound examination. There was a very high intervention rate in the fatal group, with higher NACA scores indicating increased illness severity, which is consistent with previous findings [7]. The most frequently performed critical care intervention was endotracheal intubation, which is reasonable from a pathophysiological perspective and in line with previous studies on pediatric drowning incidents [7]. Studies show that intubation is a feasible intervention following a submersion incident despite the lack of data to support a specific airway management strategy [5, 37]. Without data supporting an alternative strategy, adopting the Advanced Life Support Task Force recommendations to restore ventilation and circulation seems reasonable [38]. This study found an association between 30-day mortality and intraosseous cannulation and the use of an automated chest compression device, suggesting confounding by indication through disease severity bias, as patients with severe drowning may be more likely to receive these interventions, as observed in other studies [39]. Our results could be interpreted likewise.

According to our data, HEMS physicians need to be confident with the prehospital care of critically ill drowning patients, including airway management, intraosseous cannulation, use of an automated chest compression device, and ultrasound. Even though children accounted for a minority of the drowning population treated by the nationwide Danish Air Ambulance, this study supports the findings from Germany that HEMS physicians need to be confident with the prehospital care of drowned children of all ages [22].

### Methods compared to other studies

This study is the first to confirm using the Danish Drowning Formula developed by Breindahl et al.[12] to identify a nationwide cohort of drowning patients from the Danish Air Ambulance database. As previously speculated, this novel method of drowning identification shows a promising potential to improve the quality of future drowning research by searching high-quality databases linked to each patient’s unique civil registration number [12]. The two current studies involving the Danish Drowning Formula report on two distinct subpopulations of fatal and non-fatal drowning (those with OHCA and those treated by the Danish Air Ambulance, respectively) and can hardly be compared. More studies are needed to identify all fatal and non-fatal drowning incidents in Denmark.

### Strengths

The Danish Air Ambulance database used in this study is well-described. It provides high data quality in uniform data reporting and a high degree of data completeness (95.8–99.9% for variables concerning injury severity, prehospital diagnostics, and critical care interventions) [6, 10, 19]. Nationwide data inclusion from the Danish Air Ambulance provides a complete picture of the Danish subpopulation of drowning patients treated by a physician-staffed HEMS. This may allow for subgroup analyses and comparisons with other studies.

The positive predictive value of drowning incidents was 71% when the Danish Drowning Formula was applied to the missions with relevant primary or secondary diagnosis codes in the Danish Air Ambulance database. This adds to the evidence that text-search algorithms (e.g., Danish Drowning Formula) may provide a cost-effective solution towards future drowning identification. It was impossible to calculate the sensitivity, specificity, and negative predictive value of the Danish Drowning Formula in this study, as we did not validate the missions excluded by it and therefore have no information on the true prevalence. However, studies are ongoing to clarify these measures on a larger, unselected cohort and optimise the Danish Drowning Formula to increase its applicability for drowning identification in the future.

### Limitations

This study has some limitations. We applied the text-search algorithm within the relevant diagnosis codes in the Danish Air Ambulance database. Therefore, some drowning patients registered with other diagnosis codes may have been lost in this process. Furthermore, drowning was only registered in 45% and 23% of primary and secondary diagnosis codes, respectively, resulting in 26 patients (32%) not having a primary or secondary diagnosis code of drowning. This adds to the evidence that the International Classification of Diseases 10th Edition’s drowning codes are inappropriate for drowning research as they are unreliable [40–42].

Various limitations are linked to the retrospective design of this study. First, analyses on the effect of critical care interventions are complex due to confounding by indication, meaning the most critically ill patients may require critical care interventions more frequently. Second, despite limited missing data in the Danish Air Ambulance database, missing data may introduce a selection bias when providing lifesaving treatment and fast transport for critically ill patients. Third, drowning patients were identified based on the prehospital data in the Danish Air Ambulance database. We cannot determine the circumstances relating to the drowning event or the actual causes of death. Fourth, there were 12,360 missions without patient encounters for various reasons, including bad weather conditions in 3,015 missions. Bad weather conditions may increase the risk of drowning in natural environments [43], and these patients may have been transported by ground-based EMS or the Royal Danish Air Force’s Search And Rescue helicopters [9]. The latter are used for missions at sea and were not included in this study as they are reported in a separate system [9].

## Conclusion

The Danish Air Ambulance rarely attends to drowning patients, but those treated are severely ill, with prehospital and 30-day mortality rates of 33% and 67%, respectively. Drowning patients treated by the Danish Air Ambulance frequently require critical care interventions, particularly endotracheal intubation, the use of an automated chest compression device, intraosseous cannulation, and ultrasound examination.

## Data Availability

The datasets used and analysed during the current study are available from the corresponding author upon reasonable request.

## Abbreviations

EMS: Emergency Medical Service
HEMS: Helicopter Emergency Medical Service
ICD: International Classification of Diseases
IQR: Interquartile range
Min: Minutes
NACA: The National Advisory Committee for Aeronautics (NACA) severity score
OHCA: Out of Hospital Cardiac Arrest
ROSC: Return Of Spontaneous Circulation
STROBE: Strengthening the Reporting of Observational Studies in Epidemiology
WHO: World Health Organisation
